# Transvaginal natural orifice transluminal endoscopic surgery versus conventional vaginal surgery for sacrospinous ligament fixation of apical compartment prolapse: a retrospective analysis

**DOI:** 10.1186/s12893-023-01921-y

**Published:** 2023-01-28

**Authors:** Lu Huang, Jie Yu, Yan Li, Zhao-Lin Gong, Dan Feng, Li He, Yong-Hong Lin

**Affiliations:** Department of Gynecology, Chengdu Women’s and Children’s Central Hospital, School of Medicine, University of Electronic Science and Technology of China, Chengdu, Sichuan People’s Republic of China

**Keywords:** Transvaginal natural orifice transluminal endoscopic surgery, vNOTES, Conventional vaginal surgery, Sacrospinous ligament fixation, Pelvic organ prolapse

## Abstract

**Background:**

To objectively assess the safety, feasibility, advantages, and disadvantages of transvaginal natural orifice transluminal endoscopic surgery (vNOTES) versus conventional vaginal (CV) surgery for sacrospinous ligament fixation (SSLF).

**Methods:**

We retrospectively analyzed the data of patients who underwent hysterectomy for SSLF via vNOTES or CV surgery due to apical compartment prolapse between April 2019 and April 2020 at our hospital. The patients were classified into the vNOTES group (n = 31) and CV surgery group (n = 51) based on surgical approach and their general characteristics and perioperative outcomes compared.

**Results:**

The two groups had similar general characteristics. The anatomical success and bilateral salpingo-oophorectomy rates were higher in the vNOTES than CV surgery group, while the postoperative stay was shorter in the vNOTES than CV surgery group. All differences were statistically significant. However, there were no statistically significant intergroup differences in operation time, bilateral salpingectomy rate, colporrhaphy rate, postoperative visual analog scale score, estimated blood loss, hemoglobin decrease at 72 h postoperative, maximum body temperature at 72 h postoperative, complication rate, buttock pain, or Pelvic Floor Impact Questionnaire-7 and Pelvic Floor Distress Inventory Questionnaire-20 scores at 1 year postoperative.

**Conclusions:**

VNOTES for SSLF was safe and feasible and resulted in superior objective and subjective outcomes versus CV surgery for SSLF. These findings suggest that vNOTES could be an alternative to CV surgery for SSLF.

## Background

Pelvic organ prolapse (POP) is the downward descent of the female bladder, uterus, or post-hysterectomy vaginal cuff and the small or large bowel that results in protrusion of the vagina, uterus, or both [1]. Studies report that a woman`s lifetime risk to undergo surgery for POP is 11–20% [2, 3]. Although consensus is lacking regarding the superior surgical approach, the most accepted options for apical compartment prolapse are downward displacement of the vaginal apex, uterus, or cervix, vaginal sacrospinous ligament fixation (SSLF), uterosacral ligament suspension, and abdominal sacrocolpopexy/sacrohysteropexy [4–6].

Abdominal sacrocolpopexy is considered the gold standard for the surgical treatment of apical compartment prolapse [7], but it also features some unique complications, such as mesh exposure. Therefore, SSLF is recognized as the conventional surgical method. In 1968, Richter described transvaginal SSLF as a vaginal approach to apical prolapse [8]. SSLF was previously performed through the vagina; however, there were some associated problems, such as a narrow working space and limited visual field, which made the procedure more difficult.

With the development of laparoscopic technology, endoscopic procedures of the pelvis have become more popular. Transvaginal natural orifice transluminal endoscopic surgery (vNOTES) is a new endoscopic surgical concept that combines transvaginal surgery with laparoendoscopic single-site surgery [9]. VNOTES has been performed in many gynecologic surgeries, such as adnexal surgery [10], hysterectomy [11], myomectomy [12], sacrocolpopexy [13], and uterosacral ligament suspension [14], and its feasibility has been confirmed. However, the safety and feasibility of SSLF have not yet been confirmed.

Here we retrospectively analyzed the data of patients who underwent vNOTES and conventional vaginal (CV) SSLF due to apical compartment prolapse to objectively assess the safety, feasibility, advantages, and disadvantages of vNOTES versus CV surgery for SSLF.

## Methods

This retrospective cohort study was approved by the Institutional Review Boards of Chengdu Women and Children’s Central Hospital [No. B2020(19)]. We first performed vNOTES for SSLF in April 2019. We retrospectively analyzed the data of patients in our hospital who underwent hysterectomy for SSLF via vNOTES or CV surgery due to apical compartment prolapse between April 2019 and April 2020. The inclusion criteria were as follows: (1) uterine prolapse was defined and staged as stage ≥ 2 on the Pelvic Organ Prolapse Quantification (POP-Q) system; (2) POP of any POP-Q stage treated non-surgically with unalleviated symptoms; (3) completed the 1-year follow-up; and (4) agreed to undergo SSLF as surgical treatment. Exclusion criteria were as follows: (1) prior pelvic reconstructive surgery; (2) prior hysterectomy; (3) acute infection of the reproductive system or other parts; (4) mental, psychiatric, neurological diseases, gynecological malignancy, or other diseases and inability to tolerate surgery or anesthesia; and (5) desiring future fertility.

A total of 82 patients were included in this study. First, we informed all patients about the potential benefits and risks of vNOTES and CV surgery for SSLF. Before providing informed consent, all patients were self-selected undergoing vNOTES or CV surgery for SSLF. The patients were then classified into the vNOTES (n = 31) and CV surgery (n = 51) groups.

In addition to SSLF, all patients underwent hysterectomy with salpingectomy or salpingo-oophorectomy; anterior colporrhaphy, posterior colporrhaphy, or both were performed as needed. No concomitant incontinence surgery was performed.

### Preoperative preparation

Patients in both groups were administered sodium phosphate oral solution for bowel preparation the day before the operation, iodophor scrubbing of the vagina twice the day before the operation, and a cefmetazole 1 g intravenous infusion 30 min before the operation to prevent infection.

### Surgical process

**Fig. 1:**
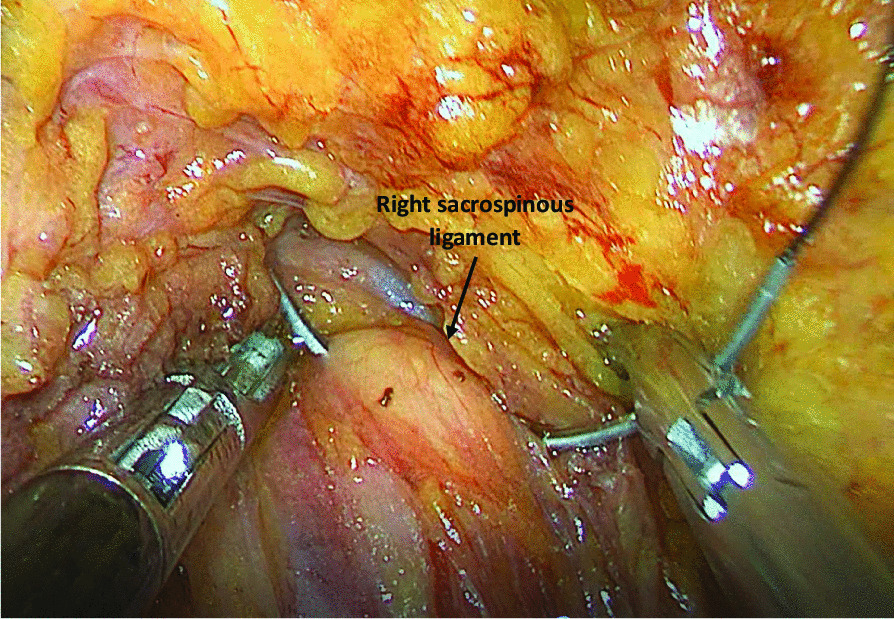
Suture the right sacrospinous Ligament

VNOTES group: (1) The patient was placed in the lithotomy position. After general anesthesia and endotracheal intubation were secured, a Foley catheter was inserted; (2) A horizontal incision was made in the posterior fornix vaginal wall and blunt dissection was performed to access the right paravaginal space; (3) A disposable multiple-instrument access port (HK-TH-60.4TY; Beijing Aerospace Kadi Technology Development Institute) was inserted into the space and 14 mmHg of CO_2_ was insufflated through the port; (4) Under direct guidance of a laparoscope (10-mm, 30° endoscope; Karl Storz GmbH & Co. KG, Tuttlingen, Germany), we continued to separate the paravaginal space until the sacrospinous ligament was revealed, and in this process, due to the lack of anatomical indication, we placed a finger into the rectum to indicate the ischial spine if necessary; (5) The sacrospinous ligament was figure-eight sutured at the point 1.5–2 cm medial to the ischial spine with a 1# Surgilon coated braided nylon nonabsorbable suture. The suture was held and left untied until other procedures were completed (Fig. 1); (6) We then performed a vNOTES hysterectomy with a salpingectomy or salpingo-oophorectomy as described previously by Su [15]; and (7) The vaginal cuff was then closed. Before finishing the vaginal cuff closure, we sutured the apex of the right vaginal wall to the sacrospinous ligament using a previously untied non-absorbable suture.

CV surgery group: Steps (1) and (2) were the same as in the vNOTES group; (3) A blunt finger dissection was used to expose the ischial spine; (4) The sacrospinous ligament was touched. Two Breisky specula were positioned and figure-eight sutured in two finger breadths medial to the ischial spine with a 1# Surgilon coated braided nylon nonabsorbable suture. A conventional vaginal surgical needle holder and an ordinary suture needle (1/2, 37 mm) were used; (5) The suture was held and left untied until the other procedures were completed; (6) A vaginal hysterectomy was performed; and (7) This step was similar to that in the vNOTES group.

Operative time was defined as the time from incision until the completion of the operation and included the operative time of all procedures: hysterectomy, SSLF, and any other concomitant procedures.

All patients in both groups used a patient-controlled analgesia pump to relieve postoperative analgesia for 48 h, and the Foley catheter was removed at 48 h postoperative. The patients met three criteria for discharge: (1) remained afebrile for at least 24 h; (2) showed no evidence of surgical complications; and (3) normal routine blood test results.

All surgeries were performed by experienced gynecologists with more than 10 years of experience performing laparoscopic and transvaginal surgeries.

### Data collection

We collected perioperative data, including age, height, weight, and reproductive history; preoperative POP-Q stage, previous operation history, operation time, estimated blood loss, preoperative and 72 h postoperative hemoglobin (Hb) level; postoperative length of stay, body temperature, and visual analog scale (VAS) score; intra- and post-operative complications within 1 month; and POP-Q stage, functional outcomes, and buttock pain at 1 year postoperative.

The VAS score was used to evaluate postoperative pain with a score of 0–10. The higher the score, the higher the pain level; that is, a score of 0 indicated painless, while a score of 10 indicated very painful.

Surgical complications were classified according to Clavien-Dindo classification [16].

This study defined anatomic success as a POP-Q stage ≤ 1 in any compartment at 1 year postoperative.

Functional outcomes were evaluated by the translated Pelvic Floor Impact Questionnaire-7 (PFIQ-7) and Pelvic Floor Distress Inventory Questionnaire-20 (PFDI-20) [17].

### Statistical analysis

SPSS version 26.0 software (SPSS Inc., Chicago, IL, USA) was used for the statistical analysis. Measurement data are expressed as mean ± standard deviation, while continuous data are expressed as frequency and percentage. The intergroup differences were compared using two independent sample *t*-tests or chi-squared tests, with values of P < 0.05 indicating statistical significance.

## Results

The two groups had similar characteristics. There were no significant intergroup differences in terms of age, body mass index, number of pregnancies, number of deliveries, number of menopausal patients, number of previous surgeries, or POP-Q stage (Table 1).

**Table 1:** Characteristics of patients

	vNOTES group(n = 31)	CV group (n = 51)	t/*χ*^2^	P
Age(year, $\overline{\chi }$ ± s)	61.42 ± 8.969	64.98 ± 7.339	− 1.957	0.054
BMI	24.43 ± 3.56	23.60 ± 2.72	1.196	0.235
Gravidity(times, $\overline{\chi }$ ± s)	3.81 ± 1.515	3.90 ± 1.889	− 0.239	0.812
Parity(times, $\overline{\chi }$ ± s)	1.68 ± 0.832	2.06 ± 0.988	− 1.796	0.076
Vaginal birth (n, %)	31(100)	50(98.0)	0.615	1.000
Cesarean section (n, %)	0(0)	1(2.0)		
Menopause (n, %)				
Yes	24(77.4)	39(76.5)	0.010	0.921
No	7(22.6)	12(23.5)		
Previous operation history (n, %)				
Yes	2(6.5)	4(7.8)	0.055	0.814
No	29(93.5)	47(92.2)		
POP-Q stage for uterus (n, %)				
I	2(6.5)	1(2.0)	1.270	0.736
II	7(22.6)	14(27.5)		
III	21(67.7)	34(66.7)		
IV	1(3.2)	2(3.9)		

BMI, body mass index

Table 2 shows the perioperative outcomes of the groups. Surgery was completed in all 82 patients without the need to switch to conventional laparoscopy or laparotomy. The rate of bilateral salpingo-oophorectomy was significantly higher in the vNOTES group than in the CV surgery group. The postoperative stay was significantly shorter in the vNOTES group than in the CV surgery group. There were no statistically significant intergroup differences in operation time, bilateral salpingectomy rate, colporrhaphy rate, postoperative VAS score, estimated blood loss, decrease in Hb level at 72 h postoperative, maximum body temperature at 72 h postoperative, or complication rate.

**Table 2:** Surgical outcomes of both groups

	vNOTES group (n = 31)	CV group (n = 51)	t/*χ*^2^	P
Operation time(min, $\overline{\chi }$ ± s)	136.58 ± 37.39	127.73 ± 47.77	0.880	0.381
Bilateral salpingectomy (n, %)	14(45.2)	30(58.8)	1.447	0.229
Bilateral salpingo-oophorectomy (n, %)	17(54.8)	10(19.6)	10.836	0.002
Concurrent colporrhaphy (n, %)	19(61.3)	41(80.4)	3.584	0.058
Concurrent anterior colporrhaphy	19(61.3)	36(70.6)	0.755	0.385
Concurrent posterior colporrhaphy	11(35.5)	28(54.9)	2.915	0.088
VAS scores 12 h post-operation($\overline{\chi }$ ± s)	2.39 ± 0.56	2.49 ± 0.76	− 0.656	0.514
VAS scores 24 h post-operation($\overline{\chi }$ ± s)	1.52 ± 0.57	1.76 ± 0.59	− 1.882	0.064
VAS scores 48 h post-operation ($\overline{\chi }$ ± s)	1.23 ± 0.62	1.10 ± 0.50	0.975	0.334
Estimated blood loss(ml, $\overline{\chi }$ ± s)	82.52 ± 28.56	92.14 ± 47.02	− 1.028	0.307
Hb decrease 72 h post-operation (g/L, $\overline{\chi }$ ± s)	20.48 ± 9.91	22.55 ± 7.12	− 1.012	0.316
Maximum body temperature 72 h post-operation(℃, $\overline{\chi }$ ± s)	37.03 ± 0.35	37.12 ± 0.49	− 0.963	0.339
Postoperative stay(days, $\overline{\chi }$ ± s)	4.81 ± 1.25	6.29 ± 2.38	− 3.219	0.002
Complications(n, %)				
No	26(83.9)	45(88.2)	1.315	0.686
Grade I	5(16.1)	5(9.8)		
Grade II	0(0)	1(2.0)		

VAS, visual analogue scale

Table 3 shows the patients` quality of life and POP-Q stage at 1 year postoperative. The anatomic success rate of the vNOTES group was significantly higher than that of the CV surgery group. There were no statistically significant intergroup differences in buttock pain or PFIQ-7 or PFDI-20 scores at 1 year postoperative. However, the mean Pelvic Organ Prolapse Impact Questionnaire-7 and Pelvic Organ Prolapse Distress Inventory-6 score of the vNOTES group was significantly lower than that of the CV surgery group.

**Table 3:** The patients’ quality of life and POP-Q stage 1 year post-operation

	vNOTES group (n = 31)	CVgroup (n = 51)	t/*χ*^2^	P
Quality of life 1 year post-operation ($\overline{\chi }$ ± s)				
PFIQ-7	23.20 ± 8.32	25.86 ± 6.19	− 1.659	0.101
UIQ-7	11.06 ± 6.55	11.30 ± 5.13	− 0.183	0.855
POPIQ-7	5.99 ± 2.45	8.03 ± 2.78	− 3.369	0.001
CRAIQ-7	6.14 ± 3.06	6.54 ± 2.33	− 0.613	0.543
PFDI-20	47.88 ± 11.67	52.21 ± 8.07	− 1.812	0.076
UDI-6	19.35 ± 4.51	21.00 ± 4.83	− 1.527	0.131
POPDI-6	13.31 ± 4.22	15.08 ± 3.66	− 2.002	0.049
CRADI-8	15.22 ± 4.25	16.13 ± 4.01	− 0.963	0.339
Buttock pain 1 year post-operation(n, %)				
Yes	23(74.2)	40(80.0)	0.373	0.541
No	8(25.8)	10(20.0)		
POP-Q stage ≤ I 1 year post-operation(n, %)	23(74.2)	21(41.2)	10.646	0.005

PFQI-7, pelvic floor impact questionnaire short form; UIQ, urinary impact questionnaire; POPIQ, pelvic organ prolapse impact questionnaire; CRAIQ, colorectal-anal impact questionnaire; PFDI-20, pelvic floor distress inventory short form; UDI, urinary distress inventory; POPDI, pelvic organ prolapse distress inventory; CRADI, colorectal-anal distress inventory

## Discussion

Our study was designed to assess the safety and feasibility of vNOTES for SSLF and determine whether it is superior to CV surgery for SSLF. This study confirmed that vNOTES for SSLF was safe and feasible and superior to CV surgery in terms of a shortened hospital stay, improved anatomic success rate, and improved symptoms of POP.

Previous reports reported different anatomical success rates of POP surgery. Different studies used different definitions of success [18]. Kowalski et al. [19], in a systematic review, Kowalski et al. reported that the most commonly used anatomical definitions were POP-Q stage ≤ 1, Baden-Walker stage ≤ 1, and maximal vaginal descent ≤ 0 (i.e., prolapse at or above the hymen). A randomized controlled study of SSLF defined anatomic success as no apical descent greater than one-third of vaginal canal or anterior or posterior vaginal wall beyond the hymen, and the anatomic success rate of SSLF was 60.5% after 2 years of follow-up and 40.3% after 5 years of follow-up [20, 21]. Another systematic review of SSLF found that using the criterion of an objective prolapse stage ≥ 2, the failure rates were 21.3% in the anterior compartment, 7.2% in the apical compartment, and 6.3% in the posterior compartment. The follow-up period in the review was 12–83 months. They attributed the heterogeneity of the anatomic outcomes to inconsistent definitions of success rate and different compartments of vaginal support [22]. From these studies, we believe that the heterogeneity of anatomical success rate was related to the inconsistent definition of success rate as well as the inconsistent follow-up times in various studies.

The longer the follow-up time, the higher the failure rate. In this study, we defined anatomic success as a postoperative POP-Q stage ≤ 1 in any compartment. After 1 year of follow-up, the anatomical success rates of the vNOTES group and the CV surgery group were 74.2% and 41.2%, respectively. Our study showed that the anatomic success rate was significantly higher in the vNOTES versus CV surgery group. The possible reasons for this result are as follows. First, in the CV surgery group, the suture position may not have been precise because of difficult visibility and limited operative space. In addition, because of concerns regarding vascular and nerve injuries in CV surgery for SSLF, the suture may be the superficial layer of the sacral spine ligament. Thus, fixation is difficult to achieve.

In contrast, using vNOTES, a surgeon can suture the sacrospinous ligament under direct vision, resulting in a more accurate suture position and an appropriate amount of ligament tissue. However, it is worth noting that some surgeons use suture-carrying devices [23–25], which alleviates the concern regarding suture depth and bite size. In a randomized controlled trial [25], the authors concluded that the modified technique of SSLF using the tissue anchoring system is noninferior to the traditional technique for the treatment of the apical compartment at 12-month follow-up. Therefore, whether vNOTES boasts a better anatomical success rate than CV surgery for SSLF after changing the suture device requires further study.

Anatomical success is very important for the postoperative evaluation of POP, but for an individual patient, the more important outcome of surgical success is symptom relief and improved quality of life [18]. Quality of life questionnaires are commonly used to evaluate symptom distress and patient postoperative quality of life. The PFDI and PFIQ can be used by clinicians and researchers to measure the extent to which lower urinary tract, lower gastrointestinal tract, and POP symptoms affect the quality of life of women with pelvic floor disorders [17].

In this study, we used the translated PFIQ-7 and PFDI-20 to assess patients’ postoperative symptoms and quality of life. Both questionnaires contained three evaluation dimensions: lower urinary tract, lower gastrointestinal tract, and POP symptoms. Although there was no intergroup difference in the mean overall PFIQ-7 and PFDI-20 scores, the remission of POP symptoms affecting quality of life in the vNOTES group was better than that in the CV surgery group on both questionnaires. We believe that this may be related to the precise suturing and fixation of the sacrospinous ligament by vNOTES. However, because of the lack of preoperative questionnaire scores in this retrospective study, it was difficult to determine the preoperative baseline conditions of patients in either group. Therefore, there was some degree of bias in the postoperative scoring results.

Recent studies demonstrated that ovarian serous carcinoma may originate in the distal fallopian tube; moreover, prophylactic salpingectomy has a protective effect against ovarian cancer [26]. Therefore, in this study, bilateral salpingectomy or bilateral salpingo-oophorectomy was included in the preoperative surgical procedure plan for all patients. All patients in the vNOTES group underwent bilateral salpingectomy or salpingo-oophorectomy. However, 11 patients in the CV surgery group did not complete the study due to adhesion or exposure difficulties. Aharoni et al. [14] reached a similar conclusion in a study of vNOTES versus CV hysterectomy with uterosacral ligament suspension. This result is mainly due to the wider visualization, better exposure of pelvic organs, and larger operating field in vNOTES versus CV surgery [9]. Therefore, we believe that vNOTES is more suitable than CV surgery for patients requiring simultaneous adnexal surgery.

SSLF is currently performed as day surgery in some hospitals. However, due to the older age of patients undergoing SSLF, we were worried about early discharge affecting the rehabilitation process, so we did not include day surgery patients in the analysis. In our discharge judgment, patient preference requires consideration, so our SSLF patients had a longer hospital stay than those in previous studies [25]. In our study, the postoperative stay was shorter in the vNOTES versus CV surgery group. One possible reason for this is that this was an unblinded study. The medical staff provided special care to the patients in the study group to improve their feelings. Therefore, to confirm whether vNOTES features a shortened hospital stay, further blinded studies with larger sample sizes are needed.

There were no grade 3 or higher complications in either group as well as no statistically significant intergroup difference in the incidence of grade 1 or 2 complications. These results further confirmed the feasibility of vNOTES for SSLF.

However, this study had some limitations. POP is a very complex condition because it includes both physical and functional aspects [27–29], and frequently reports disorders of sexual desire, arousal, orgasm, and pain, which can decrease the quality of life and affect the relationship between partners. As this was a retrospective study, data are lacking on patients` preoperative and postoperative sexual function, so it was impossible to assess the improvement of patients' sexual function after surgery. In future prospective studies, we will evaluate patients’ sexual function to compensate for this deficiency.

## Conclusions

Our study findings demonstrate that vNOTES for SSLF is safe and feasible. Compared with CV surgery for SSLF, it resulted in superior objective and subjective outcomes. Therefore, we believe that vNOTES could be used as an alternative to CV surgery for SSLF.

## Data Availability

The datasets used and/or analyzed during the current study are available from the corresponding author upon reasonable request.

## Abbreviations

vNOTES: Transvaginal natural orifice transluminal endoscopic surgery
CV: Conventional vaginal
SSLF: Sacrospinous ligament fixation
VAS: Visual analog scale
POP: Pelvic organ prolapse
Hb: Hemoglobin
PFIQ-7: Pelvic floor impact questionnaire
PFDI-20: Pelvic floor distress inventory
BMI: Body mass index
